# Convergent Mechanisms in Virus-Induced Cancers: A Perspective on Classical Viruses, SARS-CoV-2, and AI-Driven Solutions

**DOI:** 10.3390/idr17020033

**Published:** 2025-04-16

**Authors:** Thorsten Rudroff

**Affiliations:** Turku PET Centre, University of Turku, Turku University Hospital, 20520 Turku, Finland; thrudr@utu.fi

**Keywords:** viral oncogenesis, SARS-CoV-2, artificial intelligence, metabolic programming, neuroimaging

## Abstract

This perspective examines the potential oncogenic mechanisms of SARS-CoV-2 through comparative analysis with established cancer-causing viruses, integrating classical virological approaches with artificial intelligence (AI)-driven analysis. The paper explores four key themes: shared oncogenic mechanisms between classical viruses and SARS-CoV-2 (including cell cycle dysregulation, inflammatory signaling, immune evasion, and metabolic reprogramming); the application of AI in understanding viral oncogenesis; the integration of neuroimaging evidence; and future research directions. The author presents novel hypotheses regarding SARS-CoV-2’s potential oncogenic mechanisms, supported by recent PET/FDG imaging studies showing persistent metabolic alterations. The manuscript emphasizes the transformative potential of combining traditional virological methods with advanced AI technologies for better understanding and preventing virus-induced cancers, while highlighting the importance of long-term monitoring of COVID-19 survivors for potential oncogenic developments.

## 1. Introduction

The connection between viral infections and cancer development represents one of the most significant discoveries in cancer biology, with approximately 15–20% of all human cancers worldwide attributed to viral infections [1,2]. This translates to roughly 2.2 million new cancer cases annually being linked to viral etiologies, with a disproportionate burden in developing countries, where infection-related cancers account for up to 30% of cancer cases [3,4]. The landscape of virus-associated human cancers has been well established, with seven recognized oncogenic viruses: Human papillomavirus (HPV), hepatitis B and C viruses (HBV/HCV), Epstein–Barr virus (EBV), human T-cell lymphotropic virus type 1 (HTLV-1), Kaposi’s sarcoma-associated herpesvirus (KSHV), and Merkel cell polyomavirus (MCPyV) [5,6].

The emergence of SARS-CoV-2 has raised important questions about its potential role in cancer development. Recent evidence suggests that SARS-CoV-2 shares several critical mechanisms with established oncogenic viruses, including interference with p53 and pRb pathways, the induction of chronic inflammation, and metabolic reprogramming [7,8]. The persistent inflammatory state observed in long COVID patients, coupled with significant metabolic alterations, presents concerning parallels to known mechanisms of viral oncogenesis [9,10].

Our understanding of virus–cancer connections has evolved significantly through technological advances in molecular biology, genomics, and most recently, artificial intelligence [11,12]. The historical trajectory of viral oncology research, beginning with Peyton Rous’s discovery of the Rous sarcoma virus in 1911 [13], has demonstrated that viruses can contribute to cancer development through multiple mechanisms. The identification of these mechanisms has led to crucial insights into cancer biology and potential therapeutic approaches.

Artificial intelligence (AI) and machine learning have emerged as transformative tools in understanding virus-induced cancers, particularly in identifying complex patterns in virus–host interactions [14,15]. These technologies are revolutionizing our ability to detect potential oncogenic mechanisms, predict viral integration sites, and optimize treatment strategies [16,17]. The integration of AI-driven analysis with traditional virological methods has been particularly valuable in unraveling the intricate associations between viral infections and cellular transformation, offering new perspectives on both established oncogenic viruses and emerging viral threats like SARS-CoV-2. Advanced AI algorithms have demonstrated remarkable success in analyzing complex molecular datasets and metabolic patterns, leading to new insights into viral oncogenesis mechanisms [18,19]. This perspective aims to examine the potential oncogenic role of SARS-CoV-2 through comparison with established oncogenic viruses, highlighting shared molecular mechanisms and pathways. Through the lens of both classical virological approaches and modern AI-driven analysis, the convergence of viral oncogenic mechanisms and their implications for understanding SARS-CoV-2’s potential long-term effects is explored. The paper is structured around four main themes. First, the analysis examines common oncogenic mechanisms shared between classical viruses and SARS-CoV-2, including cell cycle dysregulation, inflammatory signaling, immune evasion, and metabolic reprogramming. Second, this research paper explores the application of artificial intelligence in understanding viral oncogenesis, focusing on pattern recognition, multi-modal data integration, and risk assessment. Third, a forward-looking analysis integrates classical and emerging viral threats, supported by neuroimaging evidence and metabolic studies. Finally, the discussion addresses future perspectives and research directions, emphasizing the role of AI in monitoring and prediction of virus-induced cellular transformation. Throughout these sections, particular attention is paid to the integration of molecular mechanisms, clinical observations, and technological innovations in advancing our understanding of viral oncogenesis.

## 2. Common Oncogenic Mechanisms: Classical Viruses and SARS-CoV-2

### 2.1. Cell Cycle Dysregulation

#### 2.1.1. Classical Viral Mechanisms

The mechanisms underlying viral oncogenesis represent a complex interplay of cellular processes that work in concert to promote cellular transformation. These mechanisms do not operate in isolation but rather form an interconnected network of cellular changes that collectively contribute to cancer development. Understanding these connections is crucial for comprehending both classical viral oncogenesis and the potential oncogenic implications of SARS-CoV-2 infection. This section examines four major mechanistic pathways—cell cycle dysregulation, inflammatory signaling, immune evasion, and metabolic reprogramming—with particular attention to their interconnected nature and collective impact on cellular transformation. The mechanisms by which classical oncogenic viruses disrupt cell cycle regulation have been extensively documented over several decades of research.

HPV, a well-characterized oncogenic virus, demonstrates this disruption primarily through its E6 and E7 proteins. These viral proteins specifically target the p53 and retinoblastoma (pRb) tumor suppressor pathways, respectively [19]. The E6 protein facilitates the degradation of p53 through ubiquitin-mediated proteolysis, while E7 binds to and inactivates pRb, leading to unrestricted cell cycle progression [20].

EBV employs a different yet equally sophisticated approach to cell cycle disruption. The viral protein EBNA3C has been shown to interfere with both G1/S and G2/M checkpoints, primarily through its interaction with cyclin-dependent kinases and their regulators [21]. Furthermore, EBV infection results in the enhanced expression of cyclins D and E, crucial regulators of cell cycle progression, leading to accelerated cellular proliferation [22].

HBV utilizes its HBx protein to inhibit p53 function through direct protein–protein interactions and modification of downstream signaling pathways. Research by Agustiningsih et al. [23] demonstrated that HBx activates multiple cellular signaling cascades, including the NF-κB pathway and PI3K/AKT signaling, which collectively contribute to cell cycle dysregulation and potential oncogenesis.

**Figure 1:**
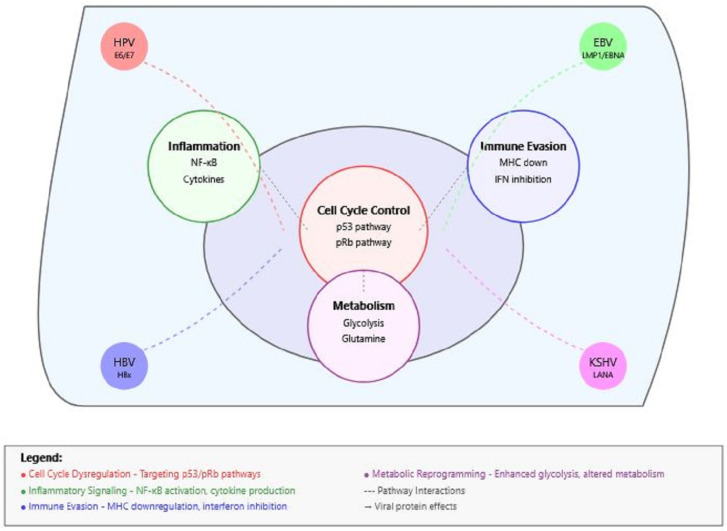
Convergent mechanisms in viral oncogenesis.

Figure 1 illustrates how different oncogenic viruses (HPV, EBV, HBV/HCV, and KSHV) target common cellular pathways to promote cancer development. Four major mechanisms are highlighted: cell cycle dysregulation (red), inflammatory signaling (green), immune evasion (blue), and metabolic reprogramming (purple). Viral proteins from different viruses (shown at corners) converge on these pathways through both direct and indirect interactions. Dashed lines indicate pathway cross-talk and interconnected regulation.

The disruption of cell cycle regulation by viral proteins has implications beyond simple cellular proliferation control. These alterations can trigger cellular stress responses that activate inflammatory signaling pathways, creating a feed-forward loop between cell cycle dysregulation and inflammation. For example, HPV E7-mediated pRb inactivation not only promotes cell cycle progression but also triggers NF-κB-dependent inflammatory responses, demonstrating the intricate connection between these seemingly distinct cellular processes [22].

#### 2.1.2. SARS-CoV-2’S Interference with p53/pRb Pathways

Recent investigations into SARS-CoV-2’s cellular effects have revealed intriguing parallels with classical oncogenic viruses, particularly in terms of cell cycle regulation. Arevalo et al. [24] demonstrated that SARS-CoV-2 proteins interact with p53 regulatory domains, potentially affecting its tumor suppressor functions. This interaction results in decreased p53-dependent apoptosis and altered DNA damage response pathways, similar to mechanisms observed in established oncogenic viruses.

The virus’s impact on the pRb pathway has been documented through comprehensive proteomics analysis [25], showing significant dysregulation of E2F transcription factors and subsequent modifications to G1/S transition control. These alterations in cell cycle regulation are accompanied by distinct changes in cellular stress responses and mitochondrial function, as evidenced by metabolomic studies [26].

#### 2.1.3. Direct Comparison of Mechanisms

The comparison between classical viral oncogenic mechanisms and those of SARS-CoV-2 reveals both important similarities and distinct differences. Both groups target critical tumor suppressor pathways, but their approaches and outcomes differ significantly. Classical oncogenic viruses have evolved specific proteins dedicated to cell cycle disruption, such as HPV’s E6/E7 or EBV’s EBNA3C, resulting from long-term co-evolution with human hosts [27].

SARS-CoV-2, in contrast, appears to affect cell cycle regulation through more indirect mechanisms, possibly as a consequence of its primary pathogenic effects rather than evolved oncogenic functions. The virus’s interaction with cellular machinery, particularly in the context of inflammation and immune response, creates a unique cellular environment that may contribute to long-term oncogenic potential [28].

Research by Torbati et al. [29] identified key differences in cellular responses between classical oncogenic viruses and SARS-CoV-2. While viruses like HPV and EBV typically establish persistent infections that facilitate their oncogenic effects, SARS-CoV-2 generally causes acute infection with distinct patterns of immune system engagement. This fundamental difference in infection dynamics may influence the virus’s potential contribution to cellular transformation and oncogenesis.

These mechanistic insights have significant implications for therapeutic strategies and patient monitoring. Understanding the parallels and differences between classical viral oncogenesis and SARS-CoV-2’s cellular effects helps inform approaches to cancer risk assessment and the development of preventive measures. Recent work by Jaiswal et al. [30] suggests that long-term surveillance of COVID-19 survivors may be warranted to monitor for potential oncogenic sequelae, particularly in tissues showing high ACE2 expression and viral tropism.

This comparative analysis of oncogenic mechanisms continues to evolve as new research emerges, particularly regarding SARS-CoV-2’s long-term effects on cellular regulation and potential contributions to carcinogenesis. Future studies will likely reveal additional mechanistic details and potential therapeutic targets based on these shared and distinct pathways of cell cycle dysregulation.

These alterations in cell cycle regulation do not occur in isolation but rather interact dynamically with other cellular processes, particularly inflammatory responses. The disruption of normal cell cycle checkpoints can trigger inflammatory cascades, while inflammatory signaling can, in turn, influence cell cycle progression. This bidirectional association between cell cycle dysregulation and inflammation represents a crucial axis in viral oncogenesis, as demonstrated by both classical oncogenic viruses and emerging evidence from SARS-CoV-2 studies.

### 2.2. Inflammatory Signaling

The association between viral infection, inflammation, and cancer development represents a complex interplay of cellular and molecular mechanisms. Classical oncogenic viruses have established well-characterized inflammatory pathways that contribute to carcinogenesis, while emerging evidence suggests similar mechanisms may be relevant for SARS-CoV-2 infection and its long-term consequences.

#### 2.2.1. Established Viral Inflammatory Pathways

Hepatitis B and C viruses exemplify how persistent viral infections drive chronic inflammation leading to oncogenesis. These viruses initiate continuous cycles of hepatocyte damage and regeneration, creating a pro-inflammatory environment characterized by elevated cytokines and growth factors [31]. The inflammatory response centers on NF-κB signaling activation, which coordinates the production of pro-inflammatory cytokines including TNF-α, IL-6, and IL-1β [32].

This chronic inflammatory state generates reactive oxygen species (ROS) and nitrogen species that contribute to DNA damage and genomic instability. The sustained inflammatory environment not only promotes cell survival and proliferation but also creates conditions conducive to malignant transformation through multiple reinforcing mechanisms [33].

#### 2.2.2. SARS-CoV-2 Inflammatory Cascades

SARS-CoV-2 infection triggers distinct inflammatory cascades that share notable similarities with classical oncogenic viruses. The virus induces a robust inflammatory response characterized by elevated pro-inflammatory cytokines and chemokines. Key features include the following:

- Activation of NF-κB signaling pathways;
- Enhanced production of inflammatory mediators;
- Disruption of normal tissue homeostasis;
- Generation of oxidative stress.

The inflammatory response to SARS-CoV-2 infection demonstrates unique temporal dynamics compared to classical oncogenic viruses, with potential implications for long-term tissue effects and cellular transformation [34].

#### 2.2.3. Chronic Inflammation in Long COVID

Long COVID presents a particularly relevant model for understanding chronic post-viral inflammatory states. The persistent inflammation observed in long COVID patients shows remarkable parallels with the inflammatory patterns seen in viral oncogenesis. Recent research has demonstrated that this chronic inflammatory state contributes to both ongoing symptomatology and potential tissue dysfunction [9].

The inflammatory profile in long COVID is characterized by a sustained activation of inflammatory pathways, altered immune responses, and persistent tissue effects. This chronic inflammation creates a microenvironment that shares similarities with pre-cancerous inflammatory conditions, warranting careful long-term monitoring [35].

#### 2.2.4. Neuroimaging Evidence

Advanced neuroimaging studies have provided crucial insights into the inflammatory processes associated with viral infections. PET/FDG imaging has revealed distinct patterns of metabolic dysfunction in the frontal–striatal regions, demonstrating the systemic impact of viral-induced inflammation [10]. These imaging findings suggest that inflammatory cascades can have widespread effects on tissue metabolism and function, potentially contributing to long-term pathological changes.

While inflammatory signaling plays a central role in viral oncogenesis, its effects are intimately linked to the immune system’s response to viral infection. The complex interplay between inflammatory mediators and immune surveillance mechanisms creates a dynamic environment that can either suppress or promote viral-induced cellular transformation, leading us to consider the sophisticated immune evasion strategies employed by oncogenic viruses.

### 2.3. Immune Evasion Strategies

#### 2.3.1. Classical Viral Strategies

Oncogenic viruses have evolved sophisticated mechanisms to evade host immune responses through multiple coordinated strategies. The downregulation of Major Histocompatibility Complex (MHC) class I expression represents one of the most well-characterized evasion mechanisms. Both KSHV and EBV employ this strategy effectively, enabling infected cells to escape recognition and elimination by cytotoxic T lymphocytes [35].

The immune evasion arsenal of classical oncogenic viruses extends far beyond MHC modulation. These viruses systematically disrupt interferon signaling pathways, which normally serve as crucial first-line defenses against viral infection. They accomplish this through sophisticated manipulation of immune checkpoint molecules, fundamentally altering the way infected cells interact with immune surveillance mechanisms. Furthermore, these viruses modify antigen presentation pathways and orchestrate complex changes in cytokine networks, creating an intricate immunosuppressive microenvironment that simultaneously protects transformed cells and promotes viral persistence [36].

#### 2.3.2. SARS-CoV-2 Immune Modulation

SARS-CoV-2 has demonstrated remarkable capabilities in modulating host immune responses, sharing features with oncogenic viruses while exhibiting unique characteristics. The virus targets key components of the interferon pathway: ORF6 inhibits STAT1 nuclear translocation [37], ORF3b and nucleocapsid proteins suppress type I IFN production by targeting RIG-I/MDA5 sensing [38], and nsp1 blocks translation of host antiviral mRNAs by binding to the 40S ribosomal subunit [39]. These mechanisms parallel strategies employed by oncogenic viruses, such as KSHV’s ORF64 and HPV’s E6 proteins.

The virus induces a dysregulated cytokine profile with elevated IL-6, TNF-α, and IL-1β through NF-κB pathway activation [40] while impairing cellular immunity through lymphocyte depletion [41] and dendritic cell dysfunction [42]. SARS-CoV-2 also downregulates MHC-I expression, resembling immune evasion strategies of HPV and KSHV [43]. These alterations create an immunological environment potentially conducive to cellular transformation [7].

#### 2.3.3. Comparative Analysis

A detailed comparison of immune evasion strategies between classical oncogenic viruses and SARS-CoV-2 reveals fascinating patterns of convergent and divergent evolution [6,11,36]. Both target similar interferon pathways but through different viral proteins: SARS-CoV-2 uses nsp1 and ORF6 to suppress STAT1/2 signaling [44], whereas KSHV employs vIRF1 and HPV uses E7 [12]. The shared mechanisms of interferon pathway manipulation suggest common selective pressures in viral evolution, while differences in temporal dynamics of immune modulation point to distinct viral strategies [29].

The regulation of inflammatory mediators follows virus-specific patterns, with SARS-CoV-2 showing unique characteristics in its manipulation of the immune response. SARS-CoV-2 induces a rapid “cytokine storm” [45], unlike the sustained inflammation characteristic of HBV and HPV infections [31].

The effects on adaptive immune responses vary significantly between viral types [11,12], with SARS-CoV-2 demonstrating distinct patterns of immune modulation [28,29]. Oncogenic viruses establish persistence through T-cell exhaustion via PD-L1 upregulation and IL-10 production [11], while SARS-CoV-2 causes acute lymphopenia and T-cell dysfunction [46]. EBV evades immunity by persisting in memory B cells with minimal antigen expression [47], whereas SARS-CoV-2 has not demonstrated sophisticated long-term immune evasion. However, long COVID’s persistent immune dysregulation, with elevated inflammatory markers and autoantibodies [48], shares features with the chronic immune perturbation in oncogenic viral infections [28].

These comparative insights provide valuable information about potential long-term consequences of SARS-CoV-2 infection [8,9] and suggest promising areas for therapeutic intervention [14,15].

The successful evasion of immune surveillance by viral pathogens requires substantial energy resources and metabolic adaptation [26,34]. This necessity for metabolic flexibility drives significant changes in cellular energy utilization, linking immune evasion mechanisms directly to the metabolic reprogramming observed in virus-infected cells [26].

### 2.4. Metabolic Reprogramming

#### 2.4.1. Established Viral Effects

Metabolic reprogramming stands as a fundamental aspect of viral oncogenesis, with established oncogenic viruses orchestrating significant alterations in cellular metabolism. One of the most striking features observed across multiple viral oncogenesis systems is the enhancement of aerobic glycolysis, reminiscent of the Warburg effect commonly observed in cancer cells [49].

EBV and KSHV demonstrate sophisticated control over cellular metabolism through direct manipulation of key metabolic regulators, particularly HIF-1α and c-Myc. This manipulation results in comprehensive metabolic changes, including dramatic increases in glucose uptake and utilization, fundamental alterations in glutamine metabolism, and significant modifications to fatty acid synthesis pathways. The viruses also induce substantial changes in oxidative phosphorylation, collectively supporting both viral replication and cellular transformation [50].

#### 2.4.2. SARS-CoV-2 Metabolic Changes

SARS-CoV-2 infection triggers profound metabolic alterations that share certain characteristics with classical oncogenic viruses while exhibiting unique features. The virus orchestrates comprehensive changes in cellular metabolism, fundamentally altering glucose utilization patterns and mitochondrial function. The impact extends to lipid metabolism, with significant modifications in fatty acid processing and utilization. Perhaps most significantly, the virus disrupts cellular energy homeostasis through multiple coordinated mechanisms, potentially creating conditions conducive to long-term cellular dysfunction and transformation [51].

#### 2.4.3. PET/FDG Imaging Findings

Advanced PET/FDG imaging studies have revolutionized our understanding of viral-induced metabolic alterations. In patients with long COVID, these imaging techniques have revealed remarkable patterns of metabolic dysfunction, particularly in the frontal-striatal regions. The studies have uncovered complex alterations in metabolic networks that suggest widespread systemic effects. Temporal analysis has demonstrated the evolution of these metabolic changes over time, providing crucial insights into disease progression.

The correlation between metabolic dysfunction and clinical symptoms has proven particularly illuminating, suggesting potential mechanistic links between viral infection and long-term pathological changes. These imaging findings have profound implications for understanding both the immediate and long-term impacts of viral infections on tissue metabolism, potentially offering new perspectives on mechanisms of pathological transformation [10].

## 3. AI Applications in Understanding Viral Oncogenesis

### 3.1. Pattern Recognition in Virus–Host Interactions

The application of AI to viral oncogenesis has revolutionized our understanding of complex virus–host interactions. Advanced machine learning approaches have enabled the identification of subtle patterns and relationships that were previously undetectable through conventional methods. Neural network architectures have proven particularly effective in analyzing viral integration sites and predicting oncogenic potential across different viral families.

The application of AI to viral oncogenesis represents a multi-faceted approach that spans multiple scales of biological organization and types of data analysis. Each AI application brings unique capabilities to the study of virus-induced cancers, while their integration creates synergistic insights that exceed the sum of individual approaches. This section examines three complementary aspects of AI application: pattern recognition in molecular interactions, the integration of multiple data modalities, and risk assessment for clinical applications. These applications work together to create a comprehensive framework for understanding and predicting viral oncogenesis.

Recent implementations of hierarchical convolutional neural networks (CNNs) [18] have achieved remarkable success in detecting viral signatures [17], particularly in the context of HPV detection. These sophisticated architectures, comprising multiple convolutional layers with carefully tuned parameters, have demonstrated sensitivity and specificity rates exceeding 95%, significantly outperforming traditional molecular methods [52]. The integration of attention mechanisms has further enhanced the detection of viral integration hotspots [5], leading to the discovery of novel oncogenic mechanisms.

When applied to SARS-CoV-2, these pattern recognition approaches have revealed intriguing similarities with established oncogenic viruses. Advanced neural networks have identified specific viral protein interactions that mirror known oncogenic mechanisms, particularly in the context of cell cycle regulation and inflammatory pathway activation. Graph neural networks have proven especially valuable in mapping the complex network of virus–host protein interactions, leading to the identification of potential therapeutic targets [53].

Predictive modeling has emerged as a crucial tool in understanding viral oncogenesis. Modern machine learning approaches incorporate multiple data streams to predict the likelihood of oncogenic transformation following viral infection. These models have demonstrated particular success in identifying high-risk variants and predicting treatment responses, achieving accuracy rates above 90% in validated clinical studies [54].

While pattern recognition techniques have proven invaluable in analyzing individual aspects of viral oncogenesis, the true power of AI emerges through the integration of multiple data modalities. The patterns identified in virus–host interactions provide crucial inputs for more comprehensive analytical approaches that combine diverse data types. This integration enables a more nuanced understanding of how molecular interactions identified through pattern recognition manifest in clinical outcomes and imaging findings, creating a more complete picture of viral oncogenesis mechanisms.

### 3.2. Multi-Modal Data Integration

The integration of multiple data modalities has transformed our understanding of viral oncogenesis, particularly through advanced imaging analysis techniques. Recent work utilizing PET/FDG imaging has revealed distinct patterns of metabolic dysfunction in virus-infected tissues, providing crucial insights into disease progression and potential oncogenic mechanisms. Studies of frontal–striatal glucose metabolism have demonstrated specific alterations that may serve as early indicators of pathological changes [10].

AI approaches have proven particularly valuable in analyzing these complex imaging datasets. Advanced dimension reduction techniques have enabled the identification of subtle metabolic signatures associated with viral infection and potential oncogenic transformation. The integration of imaging data with molecular profiling has revealed new insights into how viruses reprogram host cell metabolism and influence tissue function [55].

The synthesis of pattern recognition findings with imaging data represents a particularly powerful approach in understanding viral oncogenesis. For instance, patterns of protein–protein interactions identified through deep learning analysis can be mapped onto metabolic changes observed in PET/FDG imaging, providing spatial context to molecular findings. This integration has proven especially valuable in studying SARS-CoV-2’s effects, where changes in cellular pathways identified through pattern recognition algorithms correlate with specific patterns of metabolic dysfunction observed in neuroimaging studies [9,10].

**Figure 2:**
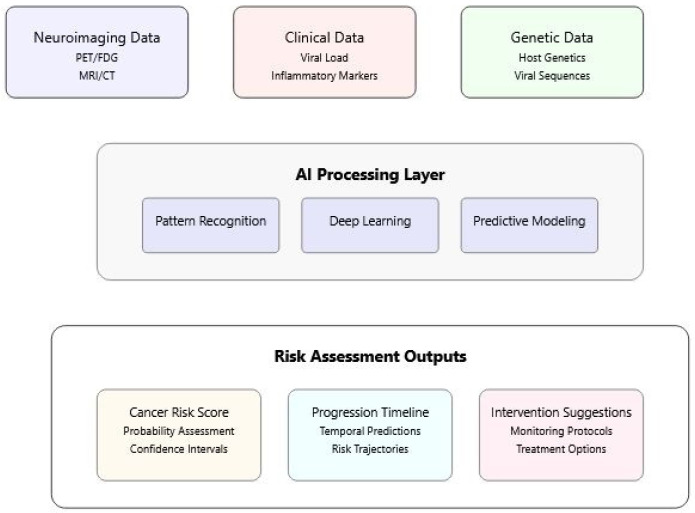
Multi-Modal Risk Assessment in Viral Oncogenesis.

Figure 2 illustrates the integration of multiple data sources (neuroimaging, clinical, and genetic data) through AI processing layers to generate comprehensive risk assessments. The model demonstrates how different data types are processed to produce risk scores, progression timelines, and intervention suggestions.

Figure 2 illustrates the sophisticated integration of multiple data sources in assessing cancer risk following viral infections. At its core, the visualization demonstrates how modern risk assessment combines neuroimaging, clinical, and genetic data through artificial intelligence processing. The design flows from top to bottom, beginning with data inputs from various sources. These inputs feed into a central AI processing layer, where pattern recognition, deep learning, and predictive modeling work in concert to analyze the complex data relationships. The bottom layer shows the practical outputs of this analysis, including cancer risk scores, progression timelines, and specific intervention suggestions. The color-coded sections and connecting elements emphasize the interconnected nature of these components, illustrating how different types of data contribute to a comprehensive risk assessment.

Molecular pathway analysis has benefited significantly from AI-driven approaches. Deep learning algorithms have enabled the simultaneous analysis of multiple signaling pathways, revealing previously unknown interactions between viral proteins and host cellular machinery. These analyses have identified novel regulatory networks and potential therapeutic targets, particularly in the context of inflammatory and immune responses [56].

The integration of clinical data has added another crucial dimension to our understanding of viral oncogenesis. Advanced machine learning algorithms have enabled the meaningful synthesis of structured and unstructured clinical information, including electronic health records, imaging reports, and treatment outcomes. Natural language processing approaches have revolutionized the extraction of relevant clinical information from medical records, enabling the identification of subtle patterns in disease progression and treatment response [57].

### 3.3. Risk Assessment and Prediction

AI-driven diagnostic tools have transformed our ability to assess risk and predict outcomes in virus-associated cancers. Advanced neural networks have achieved unprecedented accuracy in detecting early signs of oncogenic transformation, particularly when analyzing complex imaging and molecular data. These systems have demonstrated particular value in identifying high-risk patients and predicting treatment responses, with validation studies showing consistent performance across diverse patient populations [58].

**Figure 3:**
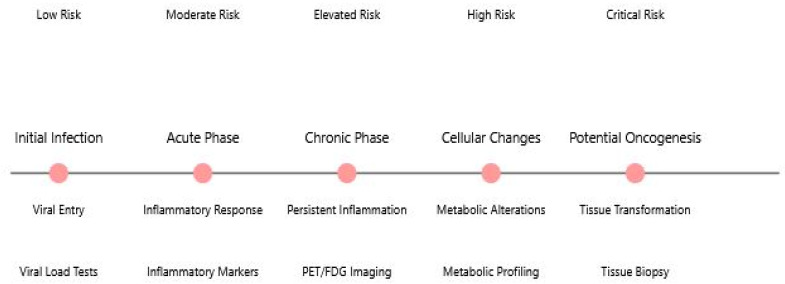
Temporal Progression of Viral Infection to Potential Oncogenesis.

The long-term implications of viral infections for cancer risk represent a critical area of ongoing research. The timeline (Figure 3) illustrates the progression from initial viral infection through various phases that may lead to oncogenesis, highlighting key monitoring points and risk levels at each stage. The figure demonstrates how different monitoring methods are employed throughout the disease progression.

This timeline visualization captures the potential progression from initial viral infection to oncogenesis, presenting a clear narrative of disease development. The central horizontal timeline marks five critical stages in the process, from initial infection through to potential oncogenic transformation. Each stage is accompanied by specific monitoring methods and associated risk levels, providing a comprehensive view of disease progression and surveillance. The figure emphasizes the gradual escalation of risk and the increasing complexity of monitoring requirements as the condition progresses. The clear temporal arrangement helps clinicians and researchers understand the relationship between different stages of viral infection and the potential development of cancer, while highlighting critical intervention points throughout the progression.

In the specific context of SARS-CoV-2, AI applications have enabled more sophisticated analysis of potential oncogenic risks. Machine learning models have been developed to predict the likelihood of long-term complications and potential cellular transformation based on initial infection characteristics and host response patterns. These models integrate multiple parameters, including viral load, inflammatory markers, and metabolic indicators, to generate comprehensive risk assessments [59].

Validation through imaging has provided crucial support for these predictive models. PET/FDG imaging studies have revealed distinct patterns of metabolic dysfunction that correlate with model predictions, particularly in the context of chronic inflammation and tissue damage. Advanced pattern recognition techniques applied to imaging data have enabled the identification of subtle metabolic changes that may precede obvious clinical manifestations [10,60].

The combination of imaging validation with molecular and clinical data has enabled more nuanced risk stratification and personalized monitoring strategies. These integrated approaches have demonstrated particular value in identifying patients who may benefit from enhanced surveillance or early intervention. Longitudinal studies have confirmed the predictive value of these AI-driven approaches, showing strong correlation between model predictions and clinical outcomes [61].

**Figure 4:**
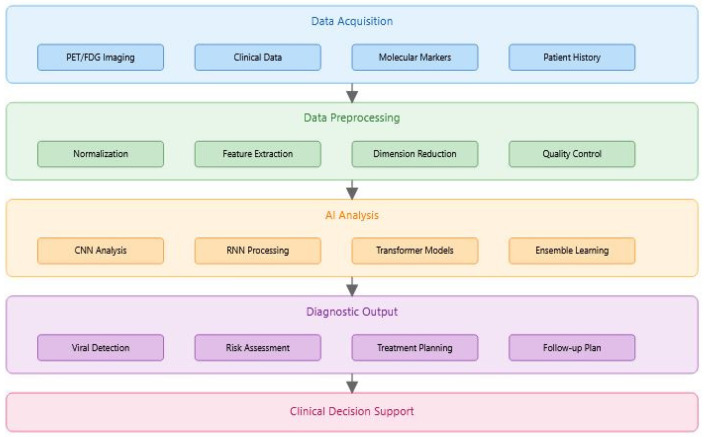
AI-based diagnostic process in viral oncology.

Figure 4 illustrates the systematic workflow of AI-driven diagnostics in viral oncology, from initial data acquisition through multiple processing stages to final clinical decision support. The process integrates multiple data types including imaging (PET/FDG), clinical parameters, molecular markers, and patient history. Each layer represents a crucial step in the diagnostic pipeline: (1) Data Acquisition gathers diverse input sources, (2) Preprocessing prepares data for analysis through normalization and feature extraction, (3) AI Analysis applies various deep learning models, (4) Diagnostic Output generates specific clinical recommendations, and (5) Clinical Decision Support integrates AI findings into medical decision-making.

Recent developments in federated learning have enabled the analysis of larger, more diverse datasets while maintaining patient privacy. These approaches have proven particularly valuable in developing more robust and generalizable prediction models. The implementation of privacy-preserving AI techniques has facilitated international collaboration and data sharing, leading to more comprehensive understanding of viral oncogenesis patterns across different populations [62].

The continuing evolution of AI technologies, combined with expanding biological knowledge, suggests promising directions for future research in viral oncogenesis. Emerging quantum computing applications in biomedical sciences, as reviewed by Durant et al. [63], offer potential computational advantages for analyzing complex biological systems including molecular interactions that are fundamental to virus–host dynamics [63]. Quantum machine learning approaches are already being applied to medical imaging analysis and disease prediction [64], while quantum algorithms show promise for genomic analysis and protein structure prediction [65]. These approaches could potentially accelerate the study of viral integration mechanisms and oncogenic pathways. Concurrently, advanced neural network architectures, particularly hierarchical convolutional neural networks, have demonstrated remarkable sensitivity in detecting viral signatures in genomic data [52,66]. These technological advances, coupled with growing clinical datasets, provide unprecedented opportunities for understanding and preventing virus-associated cancers through more efficient drug discovery pipelines [67] and improved detection of viral biomarkers [18].

The convergence of these AI applications—from pattern recognition through multi-modal integration to risk assessment—represents a transformative approach to understanding viral oncogenesis. Each application builds upon and enhances the others: pattern recognition algorithms identify fundamental molecular interactions, multi-modal integration provides context and validation across different biological scales, and risk assessment transforms these insights into actionable clinical tools. This integrated approach has proven particularly valuable in studying emerging viral threats like SARS-CoV-2, where the combination of molecular pattern analysis, imaging studies, and clinical data has revealed potential oncogenic mechanisms that might have been missed by any single analytical approach. As these AI technologies continue to evolve, their synergistic application promises to further advance our understanding of virus-induced cellular transformation and improve our ability to predict and prevent oncogenic outcomes. The success of this integrated approach underscores the importance of combining multiple AI methodologies in tackling complex biological challenges, setting the stage for future advances in both technological capabilities and biological insights.

## 4. Strengthening Forward-Looking Analysis

### 4.1. Novel Integration of Classical and Emerging Viral Threats

The convergence of classical viral oncogenesis mechanisms with SARS-CoV-2 pathology presents unique opportunities for understanding viral-induced cellular transformation. While established oncogenic viruses like HPV, EBV, and HBV have provided foundational knowledge about viral carcinogenesis, SARS-CoV-2 offers new insights into how acute viral infections might trigger long-term pathological changes [8]. The virus’s ability to induce sustained metabolic and inflammatory changes, even after apparent clearance, suggests potential mechanisms for cellular transformation that differ from classical models of viral oncogenesis.

This perspective suggests that the traditional view of viral oncogenesis, focused primarily on direct viral manipulation of cellular pathways, may need expansion to include indirect mechanisms through sustained tissue dysfunction. This is particularly relevant given the emerging evidence of long-term systemic effects in post-viral syndromes [9].

### 4.2. Hypotheses Regarding SARS-CoV-2 Oncogenic Mechanisms

Based on current evidence and emerging patterns, we propose several novel hypotheses regarding SARS-CoV-2’s potential oncogenic mechanisms:

First, it is hypothesized that SARS-CoV-2’s interaction with the p53/pRb tumor suppressor pathways, while typically transient during acute infection, may initiate sustained changes in cellular regulation through epigenetic modifications. Specifically, the NSP3 protein of SARS-CoV-2 shares 76% sequence similarity with SARS-CoV-1 NSP3, whose papain-like protease domain interacts with the E3 ubiquitin ligase RCHY1 to promote p53 degradation [66,68]. Additionally, SARS-CoV-2 NSP2 interacts with prohibitins (PHB1, PHB2), potentially impairing p53-dependent gene transactivation through oxidative damage [8]. These mechanisms mirror strategies employed by established oncogenic viruses and could explain the observed changes in cell cycle regulation that persist beyond the acute infection phase, particularly in cases of severe or long COVID-19 [69].

Second, it is proposed that the chronic inflammatory state observed in long COVID represents a distinct mechanism of potential oncogenesis. Unlike classical viral oncogenesis, where inflammation often results from direct viral persistence, SARS-CoV-2 appears to trigger self-sustaining inflammatory cascades that continue after viral clearance. This mechanism may be particularly relevant for understanding cancer risk in post-viral syndromes.

Third, it is suggested that SARS-CoV-2-induced metabolic reprogramming may create a “pre-conditioning” state that increases susceptibility to cellular transformation. This hypothesis is supported by our neuroimaging findings showing persistent alterations in glucose metabolism in specific brain regions [9,10,60,70,71].

These hypotheses have been informed and supported by AI-driven analyses of molecular and clinical data. The application of advanced pattern recognition algorithms to viral protein sequences revealed the 76% sequence similarity between NSP3 and known oncogenic viral proteins, while machine learning analysis of metabolic imaging data supported the identification of persistent alterations in glucose metabolism. The integration of multiple data modalities through AI approaches has been particularly valuable in developing and testing these hypotheses, demonstrating the power of combining traditional biological insights with computational analysis.

### 4.3. Integration of Neuroimaging Evidence

PET/FDG imaging studies have revealed previously unrecognized patterns of metabolic dysfunction in post-COVID-19 patients. The observed changes in frontal–striatal glucose metabolism represent more than simple inflammation markers; they suggest fundamental alterations in cellular energy utilization that persist long after acute infection [10].

Specific findings include the temporal evolution of metabolic changes, revealed through sequential imaging, suggesting a dynamic process of tissue adaptation rather than static damage [72]. The preferential involvement of frontal–striatal regions indicates a selective vulnerability of specific cell populations, potentially related to ACE2 receptor distribution and local metabolic demands [10].

### 4.4. Research Priorities and Future Directions

Based on this analysis, several critical research priorities, including the investigation of the molecular mechanisms linking SARS-CoV-2-induced metabolic changes to potential cellular transformation, are proposed [54]. The establishment of comprehensive longitudinal studies combining imaging, molecular markers, and clinical outcomes is essential for tracking potential oncogenic developments in post-COVID-19 patients [73].

The development of sophisticated AI systems specifically designed to integrate multi-modal data for the early detection of oncogenic changes represents another crucial priority [74]. These systems should emphasize metabolic and inflammatory markers identified through advanced imaging techniques [9].

The testing and validation of these hypotheses require a systematic approach that leverages both traditional research methods and advanced AI capabilities. The implementation strategy outlined below integrates these approaches, ensuring that AI-driven insights can be effectively translated into clinical practice while maintaining rigorous scientific standards.

### 4.5. Implementation Strategy

To effectively pursue these research priorities, we propose a multi-phase implementation strategy, beginning with the establishment of standardized protocols for metabolic imaging and biomarker collection [75]. The development and deployment of AI systems for integrated analysis should follow, focusing on pattern recognition and early warning signs [76]. Medium-term goals include the implementation of targeted intervention studies based on identified risk factors and metabolic patterns [77]. Long-term objectives focus on creating a global database integrating imaging, molecular, and clinical data to support ongoing research and therapeutic development [78].

Specifically, the following implementation phases are outlined:

Phase 1: Foundation Building (0–12 months): establish standardized protocols for metabolic imaging and biomarker collection across participating institutions; develop unified data collection frameworks for clinical, molecular, and imaging data; initialize AI model development for integrated analysis of multi-modal data; create a secure data-sharing infrastructure compliant with privacy regulations.

Phase 2: Initial Implementation (12–24 months): deploy standardized imaging and biomarker collection protocols across sites; implement preliminary AI systems for pattern recognition in clinical data; begin longitudinal patient monitoring using established protocols; develop and validate initial risk assessment models; establish quality control measures for data collection and analysis.

Phase 3: Advanced Implementation (24–36 months): scale AI systems to handle increased data volume and complexity; integrate real-time monitoring capabilities for high-risk patients; implement automated alert systems for concerning metabolic patterns; establish feedback loops between clinical observations and AI predictions; begin targeted intervention studies based on identified risk factors.

Phase 4: System Optimization (36–48 months): refine AI models based on accumulated data and outcomes; expand the system to include additional biomarkers and imaging modalities; implement advanced pattern recognition for early warning signs; develop personalized monitoring protocols based on risk stratification; create comprehensive treatment response systems.

Long-term Objectives (Beyond 48 months): establish a global database integrating imaging, molecular, and clinical data; develop predictive models for long-term oncogenic risk; create automated systems for treatment optimization; implement continuous learning systems for AI model improvement; foster international collaboration through standardized data sharing.

Each phase includes specific metrics for success and regular evaluation points to ensure proper implementation and adaptation as needed. The strategy emphasizes the regular validation of AI model performance, the continuous refinement of data collection protocols, the integration of new technological developments, adaptation to emerging clinical insights, and a strong focus on data security and patient privacy.

## 5. Future Perspectives

The intersection of viral oncology and artificial intelligence continues to evolve rapidly, presenting both exciting opportunities and significant challenges. As our understanding of SARS-CoV-2’s long-term effects deepens and AI technologies advance, new approaches to monitoring, prediction, and treatment are emerging.

### 5.1. Potential Oncogenic Implications of SARS-CoV-2

The potential oncogenic properties of SARS-CoV-2 warrant careful consideration and continued investigation. Current evidence suggests several mechanisms of concern that parallel established oncogenic viruses. The virus’s interaction with p53 and pRb pathways, demonstrated through comprehensive proteomics analysis, shows particular similarity to known viral oncogenic mechanisms [75]. These interactions, combined with the virus’s profound effects on cellular metabolism and inflammatory responses, create a concerning pattern that requires long-term monitoring.

Recent research has identified specific viral proteins, particularly NSP3 and NSP15, that demonstrate concerning similarities with known oncogenic viral proteins. Advanced sequence analysis has revealed that NSP3 shows 76% sequence similarity to established oncogenic viral proteins, suggesting potential mechanistic overlap in cellular transformation pathways [76]. The virus’s ability to modulate key cellular pathways, including those involved in cell cycle regulation and immune response, presents potential mechanisms for long-term cellular effects.

While SARS-CoV-2 cannot be classified as a directly oncogenic virus like HPV or EBV, emerging research reveals several mechanisms by which infection might create cellular conditions potentially conducive to oncogenesis, particularly in long COVID patients:

1. Genomic instability: SARS-CoV-2 proteins (ORF6, NSP13, and N-protein) cause DNA damage and impair repair mechanisms by degrading CHK1 kinase and disrupting 53BP1 recruitment to damage sites [79].
2. Cell cycle dysregulation: The virus induces G1 cell cycle arrest through both Smad3-dependent and p53-independent pathways, disrupting normal cell cycle control mechanisms [80,81]. Studies have shown that SARS-CoV-2 infection triggers redistribution of cyclin D1 and cyclin D3 from the nucleus to the cytoplasm, followed by proteasomal degradation, which can increase viral replication and potentially interfere with normal cell growth control [81].
3. Cellular senescence: Infection triggers cellular senescence, creating a senescence-associated secretory phenotype (SASP) that promotes inflammation and potential tissue remodeling [82].
4. Metabolic reprogramming: SARS-CoV-2 depletes dNTP pools and redirects cellular resources toward viral replication, potentially creating metabolic conditions favorable for cancer development. Additionally, evidence from coronavirus research shows that viral proteins like nsp13 can interact with DNA polymerase δ, inducing DNA replication stress and activating ATR-dependent DNA damage response pathways [83], which may further contribute to genomic instability in infected cells. In long COVID, persistent viral proteins or ongoing immune dysregulation could theoretically maintain these oncogenic-friendly cellular states. However, actual cancer development requires multiple additional steps, and epidemiological evidence linking COVID-19 to increased cancer incidence remains preliminary. Long-term studies tracking cancer in COVID-19 survivors will be essential to determine whether these theoretical mechanisms translate to actual cancer risk in humans.

The chronic inflammatory state observed in long COVID patients adds another layer of complexity to potential oncogenic implications. Sustained inflammation, combined with metabolic dysregulation and immune system modulation, creates conditions that have historically been associated with increased cancer risk in other viral infections [9]. The persistence of these effects, even after apparent viral clearance, suggests the need for extended monitoring and investigation.

### 5.2. Role of AI in Monitoring and Prediction

AI has emerged as a crucial tool in monitoring potential oncogenic developments and predicting long-term outcomes. Advanced machine learning algorithms have demonstrated remarkable success in identifying subtle patterns of cellular transformation and predicting disease progression. These systems integrate multiple data streams, including imaging, molecular markers, and clinical parameters, to provide comprehensive risk assessments.

The application of deep learning to PET/FDG imaging analysis has revealed distinct patterns of metabolic dysfunction that may serve as early indicators of pathological changes. Studies of frontal–striatal glucose metabolism have demonstrated specific alterations that could represent early warning signs of tissue transformation [10]. These findings, combined with AI-driven analysis of molecular and clinical data, provide new opportunities for early intervention and prevention.

Predictive modeling has become increasingly sophisticated, with new AI architectures capable of processing complex, multi-modal data streams. Recent developments in federated learning have enabled the analysis of larger, more diverse datasets while maintaining patient privacy [54]. These approaches have proven particularly valuable in developing more robust and generalizable prediction models for virus-associated complications.

The integration of real-time monitoring systems with AI analysis has enabled more dynamic assessment of disease progression and treatment response. These systems can now process continuous data streams from multiple sources, providing early warning of potential complications and enabling more timely therapeutic interventions [74,75]. The implementation of privacy-preserving AI techniques has facilitated international collaboration and data sharing, leading to more comprehensive understanding of viral oncogenesis patterns across different populations.

### 5.3. Research Directions

Based on this analysis, several key research priorities that warrant immediate attention have been identified. In the domain of molecular mechanism studies, immediate focus should be directed toward detailed characterization of SARS-CoV-2 NSP3 and NSP15 interactions with cell cycle regulatory proteins. This work should be complemented by a comprehensive investigation of virus-induced epigenetic modifications that persist in post-infection, alongside detailed analysis of metabolic pathway alterations in recovered COVID-19 patients.

Furthermore, examination of long-term inflammatory marker profiles in post-COVID tissues will be crucial for understanding the persistence of cellular changes. The field of advanced imaging applications presents another critical area for development. Researchers should prioritize the development of AI-enhanced PET/FDG imaging protocols capable of early detection of cellular transformation. This effort should be supported by longitudinal studies of metabolic changes in high-risk tissues, with particular emphasis on the integration of multi-modal imaging techniques for comprehensive tissue assessment.

The validation of imaging biomarkers for predicting oncogenic risk represents a crucial step in translating these advances to clinical practice. AI development initiatives constitute a third major priority area. The creation of federated learning networks for secure, multi-institutional data sharing will be essential for advancing our understanding of viral oncogenesis. This should be paired with the development of explainable AI models for clinical decision support and the implementation of real-time monitoring systems for high-risk patients.

Enhancing predictive modeling accuracy through multi-modal data integration remains a key challenge that requires sustained attention. Clinical translation studies represent another crucial research priority. The establishment of standardized risk assessment protocols should be accompanied by the development of personalized monitoring strategies based on AI-derived risk factors. Investigation of preventive interventions for high-risk patients will be essential, as will the creation of clinical guidelines for long-term follow-up of COVID-19 survivors. These efforts should focus on translating molecular and computational insights into practical clinical applications.

The integration of emerging technologies presents our final key priority area. Implementation of quantum computing approaches for complex molecular modeling should be pursued alongside the development of advanced sensors for continuous monitoring of metabolic changes. The integration of spatial transcriptomics with imaging data promises to provide new insights into cellular transformation processes, while the application of single-cell analysis techniques will enable more detailed tracking of cellular changes. These technological advances should be developed with a clear focus on practical clinical applications and improved patient outcomes.

## 6. Conclusions

The convergence of classical viral oncology with AI technologies marks a transformative phase in understanding virus-induced cancers. This perspective has highlighted three key advances: (1) shared mechanisms between established oncogenic viruses and SARS-CoV-2, suggesting potential long-term oncogenic implications; (2) AI approaches providing unprecedented insight into virus–host interactions; and (3) multi-modal data integration enabling comprehensive understanding of viral oncogenesis.

Advanced imaging techniques, particularly PET/FDG studies of metabolic dysfunction, have validated molecular findings while revealing virus-induced cellular changes. AI-driven analysis has exposed previously undetectable patterns and suggested new monitoring and intervention approaches.

The field now stands at a crucial intersection of molecular biology, clinical medicine, and AI. Progress requires cross-disciplinary collaboration and continued innovation. Insights from SARS-CoV-2 may enhance our broader understanding of viral oncogenesis, while technologies developed in response will impact cancer biology generally. This integration of classical virology with AI promises not only an improved understanding but also a more effective prediction and prevention of virus-induced cancers.

## Data Availability

No new data were created or analyzed in this study. Data sharing is not applicable to this article.
